# The relationship between mucosal inflammatory cells, specific symptoms, and psychological functioning in youth with irritable bowel syndrome

**DOI:** 10.1038/s41598-020-68961-9

**Published:** 2020-07-20

**Authors:** Meenal Singh, Vivekanand Singh, Jennifer V. Schurman, Jennifer M. Colombo, Craig A. Friesen

**Affiliations:** 1 Division of Gastroenterology, Hepatology, and Nutrition, Children’s Mercy Hospital, 2401 Gillham Road, Kansas City, MO 64108 USA; 2 Department of Pathology, The University of Texas Southwestern Medical Center, 1935 Medical District Drive, Dallas, TX 75235 USA

**Keywords:** Psychology, Gastroenterology, Medical research

## Abstract

Both mucosal inflammation and psychologic dysfunction have been implicated in irritable bowel syndrome (IBS). While some relationships between inflammation (mast cells and eosinophils) and depression have been reported in adults with IBS, relationships between inflammation and psychologic function have not been studied in children and adolescents. The aims of the current study were to: (1) assess densities of colonic mast cells, eosinophils, and TH17 cells in youth with IBS; and, (2) explore relationships between these cells and specific IBS symptoms and psychologic functioning. Utilizing previously obtained biopsies from the descending and rectosigmoid colons, densities were determined for mast cells, eosinophils, and TH17 cells, respectively, in 37 youth with IBS and 10 controls. In IBS patients, densities were assessed in relation to specific IBS symptoms and in relation to self-report anxiety and depression scores. In both the descending and rectosigmoid colons, densities of mast cells, eosinophils, and TH17 cells were higher in IBS patients as compared to controls. In IBS patients, rectosigmoid mast cell density was higher in those reporting pain relief with defecation. Also, in IBS patients, rectosigmoid eosinophilia was associated with higher anxiety scores and eosinophil density correlated with depression scores. In the descending colon, eosinophil and mast cell densities both correlated with depression scores. In conclusion, mucosal inflammation (mast cells and eosinophils) is associated with pain relief with defecation and with anxiety and depression in youth with IBS.

## Introduction

Chronic or recurrent abdominal pain affects a substantial proportion of children and adolescents^1,2^. The majority of youth with chronic abdominal pain will not have an identified organic disease but will report symptoms consistent with one of the functional gastrointestinal disorders (FGIDs) as defined by Rome criteria^3,4^. There are four pain related FGIDs with irritable bowel syndrome (IBS) being one of the two most common^5^. Rome IV, the most current version of Rome criteria, defines IBS by the presence of one of the following symptoms: pain related to defecation, pain associated with a change in stool frequency, or pain associated with a change in stool form^4^. IBS is further sub-categorized as IBS with predominant constipation (IBS-C), IBS with predominant diarrhea (IBS-D), mixed IBS with alternating constipation and diarrhea (IBS-M), and as unsubtyped^4,6^. Among a variety of other factors, visceral hyperalgesia, inflammation and psychosocial factors have been highly implicated in the pathogenesis of IBS^7,8^.

Inflammatory cells which have been evaluated in IBS include mast cells, eosinophils, and lymphocytes, particularly T cells. Mast cells have been highly implicated in IBS pathogenesis in both IBS-C and IBS-D^9,10^. IBS has been associated with an increase in the density of degranulating mast cells, while the density of mast cells in close proximity to enteric nerves correlates with abdominal pain severity^11^.

Eosinophils have been much less studied in the context of IBS. Although one study reported increased cecal eosinophils in adults with IBS, most studies, including one pediatric study, have found no differences in eosinophil density or in stool eosinophil protein concentrations^12–18^. While eosinophils do not appear to be increased across the population of patients with IBS, there may be a subset where eosinophils play a role. Park and colleagues reported increased colonic eosinophils in a subset (23 out of 42) of IBS patients^19^.

T lymphocytes have been implicated in both adult and pediatric IBS but TH17 cell density has not been specifically evaluated^8,20^. IL-17 has been implicated in IBS, particularly post-infectious IBS, in some but not all studies^21–23^. Increased serum IL-17 has been associated with D-IBS and related to symptom severity in one study, while serum IL-17 did not differ between IBS patients and controls in another study^24,25^. To our knowledge, density of mucosal TH17 cells has not previously been evaluated in IBS.

IBS is associated with high rates of anxiety and depression in adults which may be associated with visceral hyperalgesia and autonomic nervous system dysfunction^26^. In children with IBS, anxiety and depression correlate with abdominal pain severity^27^. In adults with IBS, mast cells density has been associated with depression^12,28^. To our knowledge, relationships between mucosal mast cells and psychological functioning have not been evaluated in children with IBS, but we have previously found an association between antral mast cells and both anxiety and depression in children with functional dyspepsia, which is another pain-associated FGID^29^. A recent study of adults with IBS in the general population found an association between eosinophil density in the transverse and sigmoid colon with depressive symptoms^30^. These studies suggest an association between colonic inflammation and psychologic functioning, at least in adults.

The goal of the current exploratory study was to further assess relationships between inflammation, symptoms, and psychologic functioning in youth with IBS which would have the potential to alter current treatment models. The aims were to: (1) assess densities of colonic mast cells, eosinophils, and TH17 cells in children and adolescents with IBS; and, (2) explore relationships between these cells and specific IBS symptoms and psychologic functioning.

## Results

### Participants

IBS patients (N = 37) ranged in age from 8 to 17 years (mean 13.8 ± 2.2 years). Seventy percent were female. All patients fulfilled Rome IV criteria for IBS. IBS subtypes consisted of IBS-D in 45.9%, IBS-M in 24.3%, IBS-C in 16.2%, and unsubtyped in 13.5%. Stools were reported to be less than daily by 13.5%, daily by 32.4%, twice daily by 27%, and three times or more daily by 27% of patients. A change in stool frequency was reported by 59.5%, a change in stool form by 67.6%, and pain relief with defecation by 51.4%. Twenty-four patients reported at least 2 of these symptoms. Of the 13 patients reporting only one symptom, pain relief with defecation was reported by 54%, a change in stool form by 31%, and a change in stool frequency by 15%.

### Cell densities

**Figure 1:**
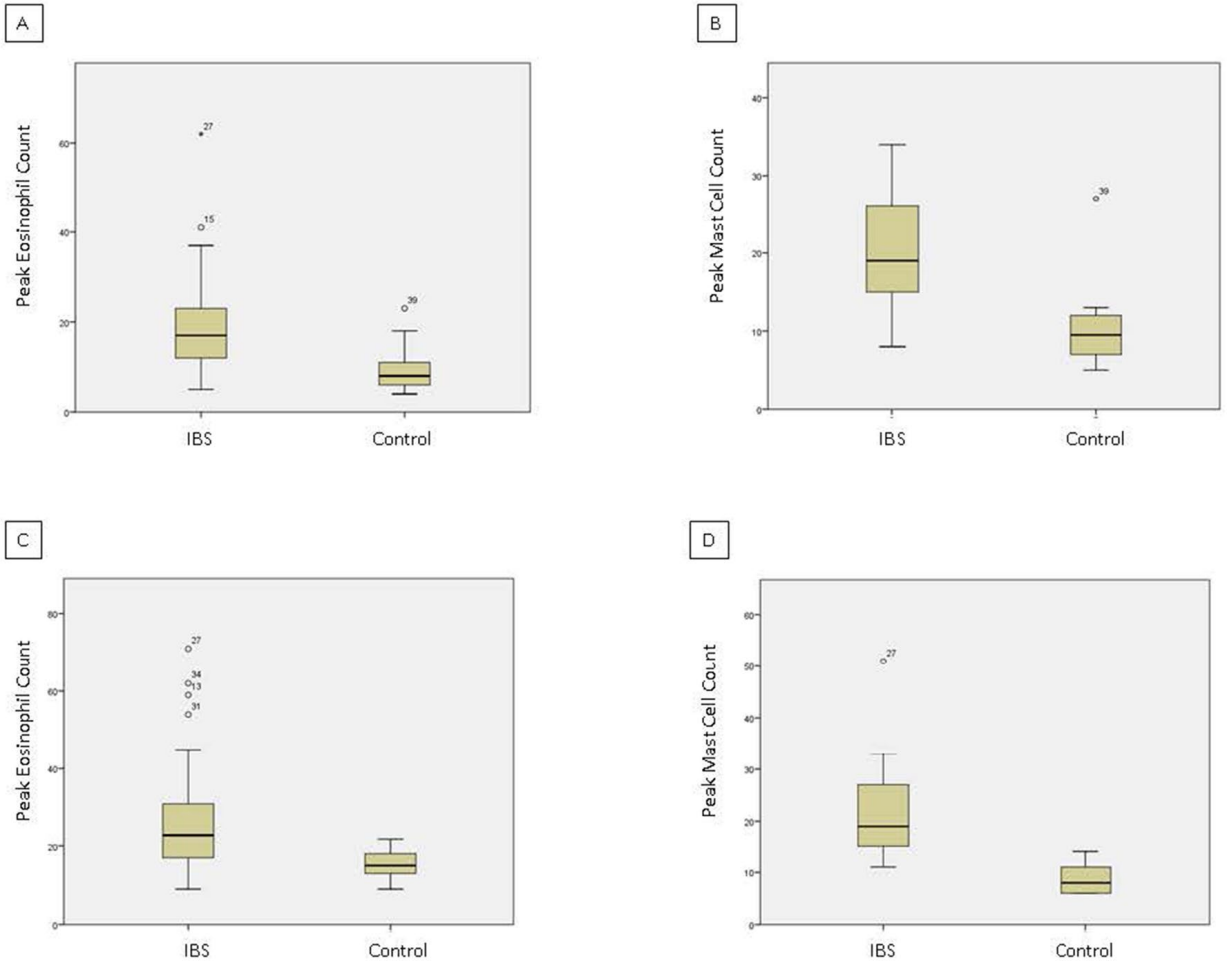
Distribution of mucosal peak eosinophils and mast cells by location for youth with IBS (n = 37) and controls (n = 10). Raw cell count data is pictured for all individuals in each study group. (**A**) Rectosigmoid peak eosinophils. (**B**) Rectosigmoid peak mast cells. (**C**) Descending colon peak eosinophils. (**D**) Descending colon peak mast cells. Peak cell counts significantly higher across location for youth with IBS versus controls (see Table 1 for p values).

Peak eosinophil density ranged from 5 to 62 in the rectosigmoid and 9 to 71 in the descending colon of IBS patients, and from 4 to 23 in the rectosigmoid and 9 to 22 in the descending colon of controls. Peak mast cell density ranged from 8 to 34 in the rectosigmoid and from 11 to 51 in the descending colon of IBS patients, and from 5 to 27 in the rectosigmoid and 6 to 14 in the descending colon of controls. Peak TH17 density ranged from 0 to 3 in the rectosigmoid and from 0 to 4 in the descending colon of IBS patients, and from 0 to 1 in the rectosigmoid and 0 to 1 in the descending colon of controls. In both locations, peak TH17 densities were 1 or less in 83.8% of IBS patients. For ease of interpretation, mean cell densities in IBS patients and controls are shown in Table 1 along with p values as determined by Mann Whitney *U*. While all statistical tests were significant between the two groups, given the lack of variability and the relative paucity of TH17 cells, they were excluded from further analysis. The distribution for cell counts is shown in Fig. 1.

**Table 1:** Mean mucosal peak eosinophil, mast cell, and TH17 densities in the rectosigmoid and descending colons of youth with IBS patients (n = 37) and controls (n = 10).

Location	Cell type	IBS patients	Controls	P value
Rectosigmoid	Eosinophils	19.0 ± 11.4	10.1 ± 6.0	0.022
Rectosigmoid	Mast cells	19.8 ± 6.4	10.7 ± 6.3	< 0.001
Rectosigmoid	TH17 cells	1.2 ± 0.5	0.5 ± 0.5	0.001
Descending	Eosinophils	26.8 ± 15.1	15.2 ± 4.0	< 0.001
Descending	Mast cells	21.5 ± 8.0	8.7 ± 2.9	< 0.001
Descending	TH17 cells	1.2 ± 0.6	0.4 ± 0.5	0.001

### Cell densities and specific symptoms

For IBS patients reporting relief of pain with defecation as compared to those reporting no relief, peak rectosigmoid mast cell density was increased while eosinophil densities did not differ. (Table 2) Neither eosinophil nor mast cell densities differed between IBS patients reporting and those not reporting a change in stool frequency, a change in stool form, diarrhea, or constipation, respectively. Likewise, cell densities did not differ between patients with IBS-C, IBS-D, IBS-M, and unsubtyped IBS.

**Table 2:** Mean mucosal peak eosinophil and mast cell densities in the rectosigmoid and descending colons of youth with IBS patients reporting pain relief with defecation (n = 19) vs. those reporting no pain relief (n = 18).

Location	Cell type	Pain relieved	Pain not relieved	P value
Rectosigmoid	Eosinophils	16.4 ± 8.3	21.7 ± 13.6	0.159
Rectosigmoid	Mast cells	22.3 ± 6.2	17.1 ± 5.7	0.011
Descending	Eosinophils	24.1 ± 12.7	29.7 ± 17.2	0.265
Descending	Mast cells	22.1 ± 6.8	20.8 ± 9.3	0.636

### Cell densities and psychologic function

Complete BASCs were available from 35 patients (95%). Anxiety scores ranged from 38 to 78 (mean 56.4 ± 11.3). Anxiety scores were < 60 in 63%, 60–69 in 23%, and ≥ 70 in 14%. Depression scores ranged from 40 to 86 (mean 50.97 ± 11.3). Depression scores were < 60 in 83%, 60–69 in 9%, and ≥ 70 in 9%. We assessed relationships utilizing clinical cut-offs for eosinophil density as these would have the potential to map onto treatment. In addition, because there is not universal agreement on density cut-offs, we assessed correlations between cell densities and psychological scores. Rectosigmoid peak eosinophils ≥ 10/hpf were associated with higher self-report anxiety (57.62 vs. 50.33; p = 0.032; Cohen’s d = 0.792). Rectosigmoid peak eosinophils ≥ 10/hpf were not associated with higher self-report depression scores (52.2 ± 12.0 vs. 44.8 ± 3.3; p = 0.147; Cohen’s d = 0.843). Peak rectosigmoid eosinophil density was correlated with self-report depression (r = 0.346; p = 0.04) Descending colon peak eosinophils ≥ 25/hpf were associated with higher self-report depression (62.7 vs. 54.7; p = 0.047; Cohen’s d = 0.690). Peak descending colon eosinophil density was correlated with self-report depression (r = 0.344; p = 0.04). Peak rectosigmoid mast cells density did not correlate with either self-report anxiety or depression scores. Peak descending colon mast cell density correlated with depression scores (r = 0.344; p = 0.04) but not anxiety scores.

## Discussion

The current study further supports a role for mast cells and possibly for eosinophils in youth with IBS. Although their density was increased over controls, TH17 cells were sparse casting doubt on any significant pathogenic role in IBS. Relationships were demonstrated in this group of patients with “pure” IBS; it is unknown whether these relationships would hold true for patients with IBS who also have overlapping FD.

Mast cells have been highly implicated in IBS where mast cells are generally increased in density, are in closer proximity to nerves, and are more likely to be degranulated^9–11,14,31^. In the current study, we also found increased mast cell density in the descending and rectosigmoid colon. Mast cell density was higher in IBS patients reporting relief of pain with defecation as compared to those not experiencing relief. Mast cell density did not differ between IBS patients who did and did not report pain associated with a change in stool form or frequency, diarrhea, or constipation. This differential association with cardinal IBS symptoms is perhaps not surprising as two previous pediatric factor analyses have not supported inclusion of pain relief with defecation in the IBS symptom complex in children and adolescents^32,33^. It is possible that symptoms may be less associated with symptom complexes and more with site-specific mast cell density. It is also possible that the lack of association with stool form and frequency is because both are under the influence of a number of other factors independent of inflammatory cells which were uncontrolled, especially diet. The mechanism accounting for pain relief with stooling is not clear but it might be explained by visceral hyperalgesia, as IBS has been associated with rectal sensitivity to distension in both adults and children^34,35^. In an animal model, visceral hyperalgesia is preceded by infiltration with mast cells and eosinophils^36^. Mast cells release mediators, primarily histamine and proteases, which can induce visceral hyperalgesia through upregulation of TRP channels, substance P, and NGF^37–39^. Mast cell-nerve interactions are directly related to pain frequency and severity in both children and adults^11,40^. There is some evidence that mast cell stabilization may decrease visceral sensitivity in adults with visceral hyperalgesia^41^.

In the current study, in addition to the increase in mast cells, eosinophil density was also increased in the descending and rectosigmoid colon of IBS patients. In adults, there has been conflicting findings related to eosinophil density in IBS^9^. Park and colleagues reported that while overall, eosinophils were not increased in IBS, there may be a subset of patients with eosinophilia^19^. De Silva and colleagues reported an increase in eosinophils in IBS but only in the cecum^13^. Previously, eosinophils have been largely unstudied in pediatric IBS except for one study where no eosinophils were seen in IBS patients or controls^15^. This is a challenging area of inquiry as true asymptomatic control data is not available because it is unethical to perform invasive procedures on children without symptoms. In presumptive normal controls, there is a wide range of “normal” eosinophil densities^42^. Given the interactions between mast cells and eosinophils, it is certainly plausible that eosinophils would be increased with mast cell activation^43–45^. We did not collect data on food allergies but is possible that the patients with eosinophilia might be ones with atypical food reactions. For example, self-reported wheat intolerance is associated with IBS and non-celiac wheat sensitivity is associated with increased duodenal and rectal eosinophils^46,47^. Utilizing provocative duodenal mucosal food challenges, atypical food allergies were identified in 70% of IBS patients with wheat accounting for 61% of these^48^. Positive reactions were associated with eosinophil degranulation without increased eosinophil density^48^.

We found significant relationships between mast cells and eosinophils, respectively, with psychological functioning. Increased mast cells in the descending colon were associated with increased depression scores. This is consistent with the relationship previously reported in adults with IBS^12,28^. It is also consistent with previous findings in children with functional dyspepsia where increased mast cells in the antrum were associated with increased anxiety and depression^29^. In the current study, elevated rectal eosinophils were associated with higher anxiety. While they were not significantly associated with depression, this may have been the result of a small sample size given the large effect size and that rectosigmoid eosinophil density was positively correlated with depression. Elevated descending colon eosinophils were associated with depression. These findings are consistent with a recent report in adults with IBS showing an association between colonic eosinophils and depression^30^. In summary, the current study indicates a relationship between mast cells and eosinophils with anxiety and/or depression. This association does not indicate cause-and-effect but raises the possibility that psychologic dysfunction may lead to inflammation or that mediators released from mast cells and eosinophils may induce anxiety and/or depression.

The current study has some limitations which should be noted. It is a relatively small sample size largely because we excluded patients with any symptoms consistent with dyspepsia and particularly under Rome IV criteria, there is a significant overlap between IBS and functional dyspepsia^5^. Given the cross-sectional design of the study, we were only able to demonstrate associations but not a cause-and-effect relationship between inflammatory cells and psychologic dysfunction. It should also be noted that cell densities are a gross measure of inflammation and may not be indicative of cell activation with subsequent release of mediators which may be of key importance in interactions between inflammation and specific symptoms or psychologic functioning.

In conclusion, we found an increase in mast cells and eosinophils in the rectosigmoid and descending colons of youth with IBS. Increases in rectosigmoid mast cells were associated with reports of pain improving with defecation, possibly suggesting a role in visceral sensitivity to distension. Mast cells and eosinophils were associated with anxiety and depression. Future studies should assess specific mediators which may explain interactions with peripheral or central neuronal function and whether visceral sensitivity or psychologic functioning are amenable to medications directed at specific mediators or mast cell stabilization.

## Methods

### Participants

The study was approved by the Institutional Review Board (IRB) Children’s Mercy Kansas City and performed in accordance with the Declaration of Helsinki. Informed consent/assent was waived by the IRB for this retrospective study. We utilized a convenience sample, retrospectively screening 250 consecutive patients presenting to an abdominal pain clinic. We identified 37 patients who had undergone colonoscopy and who were diagnosed with irritable bowel syndrome (IBS) without overlapping functional dyspepsia utilizing Rome IV criteria. We excluded patients with overlapping FD as FD has previously been shown to exhibit relationships between inflammation, symptoms, and psychologic function^29^. All patients were diagnosed by a single board-certified pediatric gastroenterologist in an abdominal pain clinic at Children’s Mercy Kansas City. All 37 patients had undergone colonoscopy with a normal gross examination. A minimum of 2 biopsies were obtained from the descending colon and the rectosigmoid colon. All patients were negative for nodularity, erosions, and ulcers. Patients ranged in age from 8 to 17 years and reported abdominal pain which occurred at least weekly for a minimum of 8 weeks.

Control specimens from 10 children (age 8–17 years) were identified from a pathology database and included patients who underwent colonoscopy for hematochezia. All had normal gross colonoscopies with the exception that they were not excluded for a single non-adenomatous polyp, fissures, or skin tags. All had biopsies with a pathology report of no diagnostic abnormality. All denied a history of abdominal pain, constipation, or diarrhea. We did not sex or age-match controls as colonic cell density does not appear to be affected by sex or age in children^49–51^.

### Measures

Questionnaires. As part of routine clinical care, all patients with IBS completed a standard medical questionnaire that contained specific questions regarding symptoms required to classify patients according to Rome IV criteria, as well as other gastrointestinal symptoms including diarrhea and constipation. IBS patients also completed the Behavior Assessment System for Children—Second Edition (BASC-2) to assess for symptoms of anxiety and depression as part of routine clinical evaluation^52^. The BASC-2 has demonstrated criterion-related and construct validity, has good internal consistency for most individual subscales, and is widely used in both clinical and research settings^52^. Standardized T scores for the self-report depression and anxiety subscales were used for the current study. A score of 60–70 is considered at clinical risk while a score ≥ 70 is considered clinically meaningful.

Histologic Evaluation. The previously obtained biopsy specimens were utilized to assess eosinophil, mast cell, and TH17 cell densities, respectively, in both the descending colon and the rectosigmoid colon. All assessments were performed by a single observer (MS) blinded to group assignment and clinical history. Hematoxylin and eosin (H&E) stained slides obtained from these patients as part of routine care were used to assess eosinophil density. Immunohistochemical (IHC) staining for tryptase and CCR6 was performed manually on formalin-fixed, paraffin-embedded tissue sections to identify mast cells and TH17 cells, respectively. Anti-human mast cell tryptase (Dako; clone AA1) was used to identify mast cells. CCR6 IHC staining (Novus Biologicals; clone 18B9E6), was used to identify TH17 cells.

To determine eosinophil density, hematoxylin and eosin stained sections were initially scanned at 10 × objective magnification to determine subjective areas of maximal density. Selecting areas of maximal density has been utilized to assess eosinophil density in children as involvement is often uneven^47,53^. Eosinophils were counted in five consecutive high-power fields (hpf; 40 × objective magnification). Likewise, mast cells tryptase-positive cells) and TH17 cells (CCR6-positive cells) were counted in five consecutive hpf after determining subjective areas of maximal involvement. All cell types were counted only in the lamina propria of the mucosa. Peak cell densities were determined in both the descending and rectosigmoid colons. All cell counts were performed by a single observer (MS).

### Statistical analysis

For each continuous variable, normality was assessed utilizing the Kolmogorov–Smirnov statistic. Continuous variables (e.g. cell densities and BASC scores) were compared between groups utilizing the Student’s *t* test when the distribution was normal and the Mann–Whitney *U* when the distribution was non-normal. One-way ANOVA was utilized for multiple group comparisons. Given the exploratory nature of this study and that we believed that the results of individual tests were important, we did not adjust for multiple comparisons as we were more concerned with Type II errors. Alternatively, we reported effect sizes where appropriate as has been recommended, calculating the cohen’s d^54,55^. Pearson correlations were assessed for eosinophil and mast cell densities, respectively, with BASC scores. A p value less than 0.05 was considered significant.

## References

[1] Chitkara DK, Rawat DJ, Talley NJ (2005). The epidemiology of childhood recurrent abdominal pain in western countries: a systematic review. Am. J. Gastroenterol..

[2] Saps M, Seshadri R, Sztainberg M, Schaffer G, Marshall BM, Di Lorenzo C (2009). A prospective school-based study of abdominal pain and other common somatic complaints in children. J. Pediatr..

[3] Rasquin A, Di Lorenzo C, Forbes D, Guiraldes E, Hyams JS, Staiano A, Walker LS (2006). Childhood functional gastrointestinal disorders: child/adolescent. Gastroenterology.

[4] Hyams JS, Di Lorenzo C, Saps M, Shulman RJ, Staiano A, van Tilburg M (2016). Functional disorders: child and adolescents. Gastroenterology.

[5] Edwards T, Friesen C, Schurman JV (2018). Classification of pediatric functional gastrointestinal disorders related to abdominal pain using Rome III vs. Rome IV criterions. BMC Gastroenterol..

[6] Mearin F, Lacy BE, Chang L, Chey WD, Lembo AJ, Simren M, Spiller R (2016). Bowel disorders. Gastroenterology.

[7] Devanarayana NM, Rajindrajith S (2018). Irritable bowel syndrome in children: current knowledge, challenges and opportunities. World J. Gastroenterol..

[8] Chumpitazi B, Shulman RJ (2016). Underlying molecular and cellular mechanisms in childhood irritable bowel syndrome. Mol. Cell. Pediatr..

[9] Burns G, Carroll G, Mathe A, Horvat J, Foster P, Walker MM, Talley NJ, Keely S (2019). Evidence for local and systemic immune activation in functional dyspepsia and the irritable bowel syndrome: a systematic review. Am. J. Gastroenterol..

[10] Bashashati M, Moossavi S, Cremon C, Barbaro MR, Moraveji S, Talmon G, Rezaei N, Hughes PA, Bian ZX, Choi CH, Lee OY, Coëffier M, Chang L, Ohman L, Schmulson MJ, McCallum RW, Simren M, Sharkey KA, Barbara G (2018). Colonic immune cells in irritable bowel syndrome: a systematic review and meta-analysis. Neurogastroenterol. Motil..

[11] Barbara G, Stanghellini V, De Giorgio R, Cremon C, Cottrell GS, Santini D, Pasquinelli G, Morselli-Labate AM, Grady EF, Bunnett NW, Collins SM, Corinaldesi R (2004). Activated mast cells in proximity to colonic nerves correlate with abdomional pain in irritable bowel syndrome. Gastroenterology.

[12] Ford AC, Talley NJ (2011). Mucosal inflammation as a potential etiological factor in irritable bowel syndrome: a systematic review. J. Gastroenterol..

[13] De Silva AP, Nandasiri SD, Hewavisehthi J, Manamperi A, Ariyasinghe MP, Dassanayake AS, Jewell DP, de Silva HJ (2012). Subclinical mucosal inflammation in diarrhea-predominant irritable bowel syndrome (IBS) in a tropical setting. Scand. J. Gastroenterol..

[14] O’Sullivan M, Clayton N, Breslin NP, Harman I, Bountra C, McLaren A, O’Morain CA (2000). Increased mast cells in the irritable bowel syndrome. Neurogastroenterol. Motil..

[15] Willot S, Gauthier C, Patey N, Faure C (2012). Nerve growth factor content is increased in the rectal mucosa of children with diarrhea-predominant irritable bowel syndrome. Neurogastroenterol. Motil..

[16] Emmanuel A, Landis D, Peucker M, Hungin APS (2016). Faecal biomarker patterns in patients with symptoms of irritable bowel syndrome. Frontline Gastroenterol..

[17] Lettesjö H, Hansson T, Peterson C, Ung KA, Ringström G, Abrahamsson H, Sinrén M (2006). Detection of inflammatory markers in stools from patients with irritable bowel syndrome and collagenous colitis. Scand. J. Gastroenterol..

[18] Kristjánsson G, Venge P, Wanders A, Lööf L, Hällgren R (2004). Clinical and subclinical intestinal inflammation assessed by the mucosal patch technique: studies of mucosal neutrophil and eosinophil activation in inflammatory bowel disease and irritable bowel syndrome. Gut.

[19] Park KS, Ahn SH, Hwang JS, Cho KB, Chung WJ, Jang BK, Kang YN, Kwon JH, Kim YH (2008). A survey about irritable bowel syndrome in South Korea Prevalence and observable organic abnormalities in IBS patients. Dig. Dis. Sci..

[20] Burns G, Carroll G, Mathe A, Horvat J, Foster P, Walker MM, Talley NJ, Keely S (2019). Evidence for local and systemic immune activation in functional dyspepsia and irritable bowel syndrome: a systematic review. Am. J. Gastroenterol..

[21] Akiho H, Ihara E, Nakamura K (2010). Low-grade inflammation plays a pivotal role in gastrointestinal dysfunction in irritable bowel syndrome. World. J. Gastrointest. Pathophysiol..

[22] Long Y, Wang W, Wang H, Hao L, Qian W, Hou X (2012). Characteristics of intestinal lamina propria dendritic cells in a mouse model of postinfectious irritable bowel syndrome. J. Gastroenterol. Hepatol..

[23] Sundin J, Rangel I, Repsilber D, Brummer RJ (2015). Cytokine response after stimulation with key commensal bacteria differ in post-infectious irritable bowel syndrome (PI-IBS) patients compared to healthy controls. PLoS ONE.

[24] Choghakhori R, Abbasnezhad A, Hasanvand A, Amani R (2017). Inflammatory cytokines and oxidative stress biomarkers in irritable bowel syndrome: association with digestive symptoms and quality of life. Cytokine.

[25] Bennet SM, Polster A, Törnblom H, Isaksson S, Capronnier S, Tessier A, Le Nevé B, Simrén M, Öhman L (2016). Global cytokine profiles and association with clinical characteristics in patients with irritable bowel syndrome. Am. J. Gastroenterol..

[26] Midenfjord I, Polster A, Sjövall H, Törnblom H (2019). Anxiety and depression in irritable bowel syndrome: exploring the interaction with other symptoms and pathophysiology using multivariate analyses. Neurogastroenterol. Motil..

[27] Hollier JM, van Tilburg MAL, Liu Y, Czyzewski DI, Self MM, Weidler EM, Heitkemper M, Shulman RJ (2019). Multiple psychological factors predict abdominal pain severity in children with irritable bowel syndrome. Neurogastroenterol. Motil..

[28] Piche T, Saint-Paul MC, Dainese R, Marine-Barjoan E, Iannelli A, Montoya ML, Peyron JF, Czerucka D, Cherikh F, Tran A, Hébuterne X (2008). Mast cells and cellularity of the colonic mucosa correlated with fatigue and depression in irritable bowel syndrome. Gut.

[29] Schurman JV, Singh M, Singh V, Neilan N, Friesen CA (2010). Symptoms and subtypes in pediatric functional dyspepsia: relation to mucosal inflammation and psychological functioning. J. Pediatr. Gastroenterol. Nutr..

[30] Andreasson, A., Walker, M.J., Agréus, L., Ljunggren, G., Schmidt, P.T. & Talley, N.J. Colonic eosinophilia is associated with current but not incident depression independent of IBS status. https://www.gastrojournal.org/article/S0016-5085(19)36913-6/pdf.

[31] Weston AP, Biddle WL, Bhatia PS, Miner PB (1993). Terminal ileal mucosal mast cells in irritable bowel syndrome. Dig. Dis. Sci..

[32] Schurman JV, Karazsia BT, Friesen CA (2017). Examination of competing diagnostic models of functional gastrointestinal disorders related to pain in children. Neurogastroenterol. Motil..

[33] Caplan A, Walker L, Rasquin A (2005). Validation of the pediatric Rome II criteria for functional gastrointestinal disorders using the questionnaire on pediatric gastrointestinal symptoms. J. Pediatr. Gastroenterol. Nutr..

[34] Van Der Veek PP, Van Rood YR, Masclee AM (2008). Symptom severity but not psychopathology predicts visceral hypersensitivity in irritable bowel syndrome. Clin. Gastroenterol. Hepatol..

[35] Di Lorenzo C, Youssef NN, Sigurdsson L, Scharff L, Griffiths J, Wald A (2001). Visceral hyperalgesia in children with functional abdominal pain. J. Pediatr..

[36] Meleine M, Accarie A, Wauters L, Toth J, Gourcerol G, Tack J, Farré R, Vanuytsel T (2019). Colonic hypersensitivity and low-grade inflammation in a spontaneous animal model for functional gastrointestinal disorders. Neurogastroenterol. Motil..

[37] Zhang L, Song J, Hou X (2016). Mast cells and irritable bowel syndrome: from the bench to the bedside. J. Neurogastroenterol. Motil..

[38] Sohn W, Lee OY, Lee SP, Lee KN, Jun DW, Lee HL, Yoon BC, Choi HS, Sim J, Jang KS (2014). Mast cell number, substance P and vasoactive intestinal peptide in irritable bowel syndrome with diarrhea. Scand. J. Gastroenterol..

[39] Xu XJ, Zhang YL, Liu L, Pan L, Yao SK (2017). Increased expression of nerve growth actor correlates with visceral hypersensitivity and impaired gut barrier function in diarrhea-predominant irritable bowel syndrome: a preliminary explorative study. Aliment. Pharmacol. Ther..

[40] Di Nardo G, Barbara G, Cucchiara S, Cremon C, Shulman RJ, Isoldi S, Zecchi L, Drago L, Oliva S, Saulle R, Barbaro MR, Stronati L (2014). Neuroimmune interactions at different intestinal sites are related to abdominal pain symptoms in children with IBS. Neurogastroenterol. Motil..

[41] Klooker TK, Braak B, Koopman KE, Welting O, Wouters MM, van der Heide S, Schemann M, Bischoff SC, van den Wijngaard RM, Boeckxstaens GE (2010). The mast cell stabiliser ketotifen decreases visceral hypersensitivity and improves intestinal symptoms in patients with irritable bowel syndrome. Gut.

[42] Kiss Z, Tél B, Farkas N, Garami A, Vincze Á, Bajor J, Sarlós P, Márta K, Erős A, Mikó A, Szakács Z, Pécsi D, Mátrai P, Hegyi P, Veres G (2018). Eosinophil counts in the small intestine and colon of children without apparent gastrointestinal disease: a meta-analysis. J. Pediatr. Gastroenterol. Nutr..

[43] Woodruff SA, Masterson JC, Fillon S, Robinson ZD, Furuta GT (2011). Role of eosinophils in inflammatory bowel and gastrointestinal diseases. J. Pediatr. Gastroenterol. Nutr..

[44] Ochi H, De Jesus NH, Hsieh FH, Austen KF, Boyce JA (2000). IL-4 and -5 prime human mast cells for different profiles of IgE-dependent cytokine production. Proc. Natl. Acad. Sci. USA.

[45] Santos J, Alonso C, Guilarte M, Vicario M, Malagelada JR (2006). Targeting mast cells in the treatment of functional gastrointestinal disorders. Curr. Opin. Pharmacol..

[46] Potter MDE, Walker MM, Jones MP, Koloski NA, Keely S, Talley NJ (2018). Wheat intolerance and chronic gastrointestinal symptoms in an Australian population-based study: association between wheat sensitivity, celiac disease, and functional gastrointestinal disorders. Am. J. Gastroenterol..

[47] Carroccio A, Giannone G, Mansueto P, Soresi M, La Blasca F, Fayer F, Lacobucci R, Catalano T, Geraci G, Arini A, D’Alcamo A, Villanacci V, Florena AM (2019). Duodenal and rectal mucosal inflammation in patients with non-celiac wheat sensitivity. Clin. Gastroenterol. Hepatol..

[48] Fritscher-Ravens A, Pflaum T, Mösinger M, Ruchay Z, Röcken C, Milla PJ, Das M, Böttner M, Wedel T, Schuppan D (2019). Many patients with irritable bowel syndrome have atypical food allergies not associated with immunoglobulin E. Gastroenterology.

[49] Lee EH, Yang HR, Lee HS (2018). Quantitative analysis of distribution of the gastrointestinal tract eosinophils in childhood functional abdominal pain disorders. J. Neurogastroenterol. Motil..

[50] Chernetsova E, Sullivan K, de Nanassy J, Barkey J, Mack D, Nasar A, El Demellawy D (2016). Histologic analysis of eosinophils and mast cells of the gastrointestinal tract in healthy children. Hum. Pathol..

[51] Grzybowska-Chlebowczyk U, Horowska-Ziaja S, Kajor M, Wiecek S, Chlebowczyk W, Woś H (2017). Eosinophilic colitis in children. Adv. Dermatol. Allergol..

[52] Reynolds CR, Kanphaus RW (1992). Behavior Assessment for Children (BASC).

[53] Saad AG (2011). Normal quantity and distribution of mast cells and eosinophils in the pediatric colon. Pediatr. Dev. Pathol..

[54] Armstrong RA (2014). When to use the Bonferroni correction. Ophthalmic. Physiol. Opt..

[55] Nakagawa S (2004). A farewell to Bonferroni: the problems of low statistical power and publication bias. Behav. Ecol..

